# Prognostic Impact of In-Hospital Use of Mechanical Cardiopulmonary Resuscitation Devices Compared with Manual Cardiopulmonary Resuscitation: A Nationwide Population-Based Observational Study in South Korea

**DOI:** 10.3390/medicina58030353

**Published:** 2022-02-27

**Authors:** Wonhee Kim, Chiwon Ahn, In-Young Kim, Hyun-Young Choi, Jae-Guk Kim, Jihoon Kim, Hyungoo Shin, Shinje Moon, Juncheol Lee, Jongshill Lee, Youngsuk Cho, Yoonje Lee, Dong-Geum Shin

**Affiliations:** 1 Department of Emergency Medicine, College of Medicine, Hallym University, Chuncheon 24252, Korea; wonsee02@gmail.com (W.K.); chy6049@naver.com (H.-Y.C.); gallion00@gmail.com (J.-G.K.); faith2love@hanmail.net (Y.C.); yong0831@naver.com (Y.L.); 2 Department of Biomedical Engineering, College of Medicine, Hanyang University, Seoul 04763, Korea; cahn@cau.ac.kr (C.A.); netlee@hanyang.ac.kr (J.L.); 3 Department of Emergency Medicine, College of Medicine, Chung-Ang University, Seoul 06974, Korea; 4 Department of Thoracic and Cardiovascular Surgery, College of Medicine, Hallym University, Chuncheon 24252, Korea; jkim@hallym.ac.kr; 5 Department of Emergency Medicine, College of Medicine, Hanyang University, Seoul 04763, Korea; seodtst@gmail.com (H.S.); jclee0221@gmail.com (J.L.); 6 Department of Internal Medicine, College of Medicine, Hallym University, Chuncheon 24252, Korea; sinjei1129@gmail.com (S.M.); blaudg@naver.com (D.-G.S.)

**Keywords:** cardiopulmonary resuscitation, heart arrest, chest compression, mechanical device

## Abstract

*Background and Objectives*: This study analyzed the prognostic impact of mechanical cardiopulmonary resuscitation (CPR) devices in out-of-hospital cardiac arrest (OHCA) patients, in comparison to manual CPR. *Materials and Methods*: This study was a nationwide population-based observational study in South Korea. Data were retrospectively collected from 142,905 OHCA patients using the South Korean Out-of-Hospital Cardiac Arrest Surveillance database. We included adult OHCA patients who received manual or mechanical CPR in the emergency room. The primary outcome was survival at discharge and the secondary outcome was sustained return of spontaneous circulation (ROSC). Statistical analysis included propensity score matching and multivariate logistic regression. *Results*: A total of 19,045 manual CPR and 1125 mechanical CPR cases (671 AutoPulse^TM^ vs. 305 Thumper^TM^ vs. 149 LUCAS^TM^) were included. In the matched multivariate analyses, all mechanical CPR devices were associated with a lower ROSC than that of manual CPR. AutoPulse^TM^ was associated with lower survival in the multivariate analysis after matching (aOR with 95% CI: 0.57 (0.33–0.96)), but the other mechanical CPR devices were associated with similar survival to discharge as that of manual CPR. Witnessed arrest was commonly associated with high ROSC, but the use of mechanical CPR devices and cardiac origin arrest were associated with low ROSC. Only target temperature management was the common predictor for high survival. *Conclusions*: The mechanical CPR devices largely led to similar survival to discharge as that of manual CPR in OHCA patients; however, the in-hospital use of the AutoPulse^TM^ device for mechanical CPR may significantly lower survival compared to manual CPR.

## 1. Introduction

Through recent bioengineering developments and its expansion to various medical fields, advanced medical equipment are being used to improve the quality of cardiopulmonary resuscitation (CPR). In airway management, video laryngoscopes significantly improve intubating performance, including improving the glottic view [1,2]. Mechanical CPR devices perform automatic chest compression (CC) for cardiac arrest patients [3]. As an alternative to manual CPR, the 2020 American Heart Association (AHA) CPR guidelines outline that mechanical CPR devices are indicated for use in special circumstances, such as for coronary angiography, the implementation of extracorporeal membrane oxygenation, or patient transportation via ambulances or helicopters [4].

Generally, mechanical CPR devices can be classified into three different CC mechanisms [5,6]. First, the AutoPulse^TM^ device (AutoPulse^®^ Resuscitation System Model 100, ZOLL^®^, Chelmsford, CA, USA) performs automatic CC using a load-distributing band with two arms that connect from the backboard [7]. When the whole rib cage, including the sternum, is compressed with the load-distributing band, blood flows to the heart and the whole body. Second, the Thumper^TM^ device (Thumper Model 1007CCMII Mechanical CPR System, Michigan instruments Inc., Grand Rapids, MI, USA) performs CC using the driving force generated by compressed oxygen with one connecting arm from the backboard [8]. The piston of the Thumper^TM^ device mimics the mechanism of manual CC and can directly compress the heart over the sternum. Third, the LUCAS^TM^ device (LUCAS^TM^2 Chest Compression System, JOLIFE AB Inc., Lund, Sweden) uses the driving force generated by electricity to perform CC using two connecting arms and a suction cup; the device can directly compress the sternum and induce active decompression via the suction cup [9].

In a recent systematic review, the AutoPulse^TM^ device led to a higher incidence of pneumothorax and subcutaneous hematoma than manual CPR; however, the LUCAS^TM^ device has been shown to be equivalent to manual CPR [10]. The difference in the CC mechanism between these mechanical CPR devices may affect patient outcomes, such as the return of spontaneous circulation (ROSC) or survival. Therefore, our aim was to investigate the prognostic impact of three mechanical CPR devices used in-hospital for out-of-hospital cardiac arrest (OHCA) patient outcomes, compared with manual CPR, using nationwide surveillance data from South Korea.

## 2. Materials and Methods

### 2.1. Study Design

This was a retrospective nationwide population-based observational study that used data from the Out-of-Hospital Cardiac Arrest Surveillance (OHCAS) database from the Korea Disease Control and Prevention Agency (KDCA) in South Korea (http://kdca.go.kr/, accessed on 1 March 2021). Over the period from 2012 to 2016, all acute OHCA patients transferred to medical institutions via emergency medical services were included in this study. Approximately 30,000 patients per year and 600 medical institutions were included. KDCA investigator visited the medical institution to review patient medical records and verify several items according to the Utstein Style and Resuscitation Outcome Consortium Project.

### 2.2. Participants

The study population included adult patients (18 years of age and older) who had been witnessed as OHCA patients between January 2012 and December 2016. The intervention group included patients who received mechanical CPR in the emergency room using one of three CPR devices (AutoPulse^TM^, Thumper^TM^, or LUCAS^TM^). The control group included patients who received manual CPR. During pre-hospital CPR, the paramedics performed manual CPR for the patients in both groups. In addition, no patients received CPR using mechanical CPR devices until their arrival to the medical institution. Exclusion criteria were: trauma, patient with ROSC prior to arrival at the medical institution, death on arrival, patients with “do not resuscitate” orders, patients under 18 years or age, and patients who were transferred to another medical institution after emergency room management.

### 2.3. Outcome Measures

The primary outcome was survival at hospital discharge. The secondary outcome was the sustained ROSC (>20 min) in the emergency room.

### 2.4. Data Extraction

We extracted covariates to compare and analyze the survival and ROSC of patients in the intervention and control groups. The results were analyzed as follows: (1) Mechanical CPR using AutoPulse^TM^ versus manual CPR; (2) mechanical CPR using Thumper^TM^ versus manual CPR; and (3) mechanical CPR using LUCAS^TM^ versus manual CPR. The covariates included prognostic factors that had been reported to be significantly related to the survival and ROSC of arrest patients in previous research [3,4,10]. More specifically, the following covariates were included and analyzed in the univariate analysis: age, sex, location of the cardiac arrest (public vs. non-public), bystander CPR, cause of arrest (cardiac vs. non-cardiac), initial cardiac arrest rhythm during CPR (shockable vs. non-shockable), transport time to the medical institution, percutaneous coronary intervention (PCI), target temperature management (TTM), pacemaker, and extracorporeal membrane oxygenation (ECMO).

### 2.5. Statistical Analyses

The categorical variables were analyzed using Pearson’s Chi-squared and Fisher’s exact tests. Continuous variables were analyzed using an independent samples t-test for parametric data and Mann–Whitney *U*-test for non-parametric data. A Shapiro–Wilk test was used to assess data normality. Propensity score matching (PSM) analysis was used to adjust for the covariates and lower their confounding effects for both the control and experimental groups. Since matching cannot be performed when there are missing values, all missing values for each variable were completely omitted prior to matching. PSM model was developed including all variables with no missing value. Matching was performed for each of the three devices separately, and 1:1 was conducted without replacement of variables. Multivariate analysis using logistic regression was additionally performed using all statistically significant covariates from the univariate analysis; any variables with *p* < 0.05 in the univariate analyses were included in the regression. Logistic regression with backward elimination was performed for all significant factors in univariate analysis. Following the stepwise elimination of factors in the regression, only the factors that optimize the model’s coefficient of determination remained. The *p*-value criterion for covariate entry was 0.2. All data analyses were performed using R (version 4.1.1, The R foundation for Statistical Computing).

### 2.6. Ethics Statement

The local ethics committee approved this study (Kangnam Sacred Heart Hospital’s Institutional Review Board No. HKS 2018-10-020); the need for informed consent was waived off due to the study’s retrospective nature and the use of anonymous clinical data. The KCDC approved the use of the data for this study. In addition, the methodology fulfilled the criteria of the Strengthening the Reporting of Observational Studies in Epidemiology checklist [11].

## 3. Results

### 3.1. Study Subject Characteristics

In this study, mechanical CPR using three different mechanical CPR devices (AutoPulse^TM^, Thumper^TM^, and LUCAS^TM^) was analyzed, in comparison with manual CPR. The specifications of the mechanical CPR devices are shown in Table 1. AutoPulse^TM^ is characterized by CC performed by a compression band, and Thumper^TM^ is characterized by CC performed by oxygen or air pressure. The compression tool in the LUCAS^TM^ device can be absorbed in the form of a cup shape, such that it adheres to the patient’s chest surface to induce active decompression.

**Figure 1:**
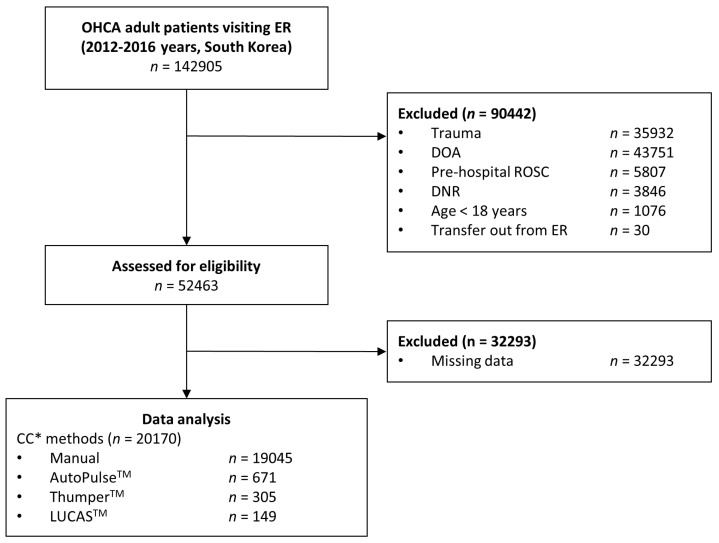
Flow diagram for the identification of relevant studies. A star (*) indicates chest compression in emergency room. OHCA, out-of-hospital cardiac arrest; ER, emergency room; DOA, dead on arrival; ROSC, return of spontaneous circulation; DNR, do not resuscitate; and CC, chest compression.

A total of 142,905 OHCA patients were registered in the database, all of which were evaluated for their eligibility for inclusion in this study. After excluding 32,293 patients with missing data, 20,170 patients were included in our analysis (Figure 1). The number of patients in each of the four comparison groups was as follows: 19,045 patients received manual CPR; 671 patients received mechanical CPR with AutoPulse^TM^; 305 patients received mechanical CPR with Thumper^TM^; 149 patients received mechanical CPR with LUCAS^TM^.

The clinical characteristics of the patients from the unmatched data in the manual and mechanical CPR groups are summarized in Supplementary Table S1. In the unmatched univariate analysis, there was a significant difference between the mechanical and manual CPR groups for some covariates. In the AutoPulse^TM^ vs. manual CPR comparison, the significant covariates were age, arrest rhythm, PCI, and pacemaker (*p* = 0.016, 0.010, 0.009, and 0.017, respectively). In the Thumper^TM^ vs. manual CPR comparison, the only significant covariate was TTM (*p* = 0.028). In the LUCAS^TM^ vs. manual CPR comparison, the significant covariates were bystander CPR and ECMO (*p* = 0.005 and <0.001, respectively). Comparing each device of mechanical CPR and manual CPR, sustained ROSC and survival at discharge were significantly higher in manual CPR (*p* of AutoPulse^TM^ = less than 0.001, 0.028, *p* of Thumper^TM^ = less than 0.001, 0.011, and *p* of LUCAS^TM^ = 0.003, 0.048, respectively).

### 3.2. Matched Univariate Analysis

For the mechanical CPR with AutoPulse^TM^ and manual CPR comparison, 1:1 PSM was applied to the three imbalanced covariates (i.e., age, arrest rhythm, PCI); the results are shown in Table 2. Both groups equally included 671 patients. Patients in the mechanical CPR with AutoPulse^TM^ group showed a higher frequency of witnessed cardiac arrest (62.9 vs. 56.6%, *p* = 0.022), TTM rates (7.0 vs. 3.3%, *p* = 0.003), and ECMO (2.8 vs. 1.0%, *p* = 0.029), compared to manual CPR. Other covariates did not significantly differ across both groups. In terms of outcomes, there was a lower rate of sustained ROSC in the AutoPulse^TM^ group (30.3 vs. 35.8%, *p* = 0.037), but there was no significant difference between groups in survival at discharge (4.9 vs. 6.3%, *p* = 0.342).

For the mechanical CPR with Thumper^TM^ and manual CPR comparison, 1:1 PSM was applied to the imbalanced covariate (TTM). Both groups included 305 patients. Patients in the mechanical CPR with Thumper^TM^ group showed a higher rate of arrest in a public location compared to the manual CPR group. Other covariates did not significantly differ across both groups. With respect to outcomes, the Thumper^TM^ group showed a lower rate of sustained ROSC compared with manual CPR (20.3 vs. 36.1%, *p* < 0.001). There was no significant difference across groups for survival at discharge (3.3 vs. 4.6%, *p* = 0.532).

For the mechanical CPR with LUCAS^TM^ and manual CPR comparison, 1:1 PSM was applied to the two imbalanced covariates (bystander CPR and ECMO). Both groups included 149 patients. There were no imbalanced covariates in either group after PSM. In terms of outcomes, the LUCAS^TM^ group showed a lower rate of sustained ROSC compared to manual CPR (27.5 vs. 46.3%, *p* = 0.001); however, there was no significant difference across groups for survival at discharge (2.7 vs. 5.4%, *p* = 0.377).

### 3.3. Matched Multivariate Analysis

**Figure 2:**
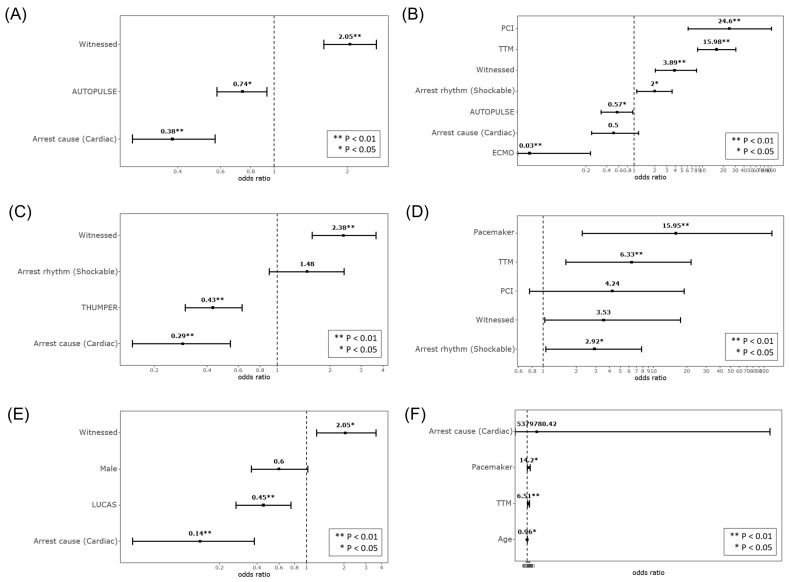
Forest plot of matched multivariate analysis for three types of mechanical CPR devices. (**A**) AutoPulse^TM^ for sustained ROSC. (**B**) AutoPulse^TM^ for survival at discharge. (**C**) Thumper^TM^ for sustained ROSC. (**D**) Thumper^TM^ for survival at discharge. (**E**) LUCAS^TM^ for sustained ROSC. (**F**) LUCAS^TM^ for survival at discharge. * was given a statistical significance of *p* < 0.05. ** was set at *p* < 0.01 indicating statistical significance.

Across all three mechanical CPR devices, the use of mechanical CPR devices, witnessed arrest, and cardiac origin arrest were common significant predictors for sustained ROSC (Figure 2 and Table 3). Witnessed arrest was significantly associated with high ROSC, but the use of mechanical CPR devices and cardiac origin arrest were associated with low ROSC. With respect to survival at discharge, TTM was the only significant predictor for high survival across all three types of mechanical CPR devices (Figure 2 and Table 4).

In the multivariate analysis of matched cases, mechanical CPR with AutoPulse^TM^ and cardiac origin arrest showed a low sustained ROSC (aOR with 95% CI: 0.74 (0.58–0.93) and 0.38 (0.26–0.57), respectively). However, witnessed cardiac arrest showed a high sustained ROSC (aOR with 95% CI: 2.05 (1.60–2.62); Table 3). Additionally, the use of AutoPulse^TM^ and ECMO was associated with lower survival at discharge (aOR with 95% CI, 0.57 (0.33–0.96) and 0.03 (0.00–0.34), respectively; Table 4). Conversely, the following factors were associated with higher survival: witnessed cardiac arrest, arrest rhythm, PCI, TTM, and ECMO (aOR with 95% CI: 3.89 (1.95–7.73), 2.00 (1.11–3.61), 24.60 (6.26–96.76), 15.98 (8.41–30.37), and 0.03 (0.00–0.34), respectively).

In the analysis between mechanical CPR with Thumper^TM^ and manual CPR, the use of Thumper^TM^ and cardiac origin arrest were significantly associated with low sustained ROSC (aOR with 95% CI: 0.43 (0.30–0.63) and 0.29 (0.15–0.54), respectively; Table 3); however, witnessed cardiac arrest was significantly associated with high sustained ROSC (aOR with 95% CI: 2.38 (1.57–3.60)). The use of the ThumperTM as a mechanical CPR device was not significantly related to survival at discharge because it was removed from the final regression model (Table 4). Three factors were significant predictors for high survival: shockable arrest rhythm, TTM, and pacemaker (aOR with 95% CI: 2.92 (1.09–7.79), 6.33 (1.75–22.98), and 15.95 (2.33–109.00), respectively).

In the analysis between mechanical CPR with the LUCAS^TM^ device and manual CPR, the use of LUCAS^TM^ and cardiac origin arrest were significantly associated with low sustained ROSC (aOR with 95% CI: 0.45 (0.27–0.75) and 0.14 (0.05–0.41), respectively; Table 3). Moreover, witnessed arrest was the only significant predictor (aOR with 95% CI: 2.05 (1.19–3.55)). The use of the LUCASTM as a mechanical CPR device was not significantly related to survival at discharge because it was removed from the final regression model (Table 4). However, young age, TTM, and pacemaker were significantly associated with high survival (aOR with 95% CI: 0.96 (0.92–1.00), 6.51 (1.65–25.74), and 14.20 (1.48–136.61), respectively).

## 4. Discussion

This study is a nationwide population-based observational study. OHCA patients who experienced three different types of mechanical CPR devices used in-hospital were compared to those who received manual CPR; their ROSC and survival rates were analyzed. In the univariate analysis after PSM, all three mechanical CPR devices showed lower sustained ROSC than that of manual CPR. Survival at discharge after the use of these mechanical CPR devices was equivalent to that of patients who received manual CPR. In the multivariate analysis for survival, in-hospital procedures such as TTM, PCI, pacemaker, and ECMO were significant prognostic factors, even in OHCA patients. These results may provide additional scientific evidence for the clinical importance of post-cardiac arrest care in OHCA patients.

There were several studies comparing mechanical CPR in hospitals against manual CPR [12,13,14,15]. Hayashida et al. utilized national data in Japan and showed that mechanical CPR was associated with significantly worse outcome [12]. This study reported the long no flow period caused by transmission to device from manual CPR, as well as the device’s lack of real-time input on depth, rate, recoil, and cycle. Several CPR devices were included in this study, however no subgroup analysis was conducted. AutoPulse^TM^ requires a long time to apply for an arrest patient due to its complex and heavy structure. Other machines, on the other hand, have the potential to reduce the time it takes to apply them to a patient. Furthermore, because the mechanisms for compression depth, speed, and decompression used by different machines differ, manual CPR must be compared independently.

In this study, the enrolled OHCA patients received manual CPR during the pre-hospital stage. All three types of mechanical CPR were in-hospital CPR performed in the emergency room. Each device has its own unique features and characteristics (Table 1). The automatic chest compression device’s compression movement is similar to the mechanism that successfully returns blood to the heart during manual chest compression [5], and each device may be classified according on the compression mechanism’s features [6]. To squeeze the entire ribcage, compress the heart, and increase blood flow, AutoPulse^TM^ uses two load distributing bands attached to the backboard [7]. Since compression depth is only 20% of chest depth, compression depth might differ depending on the chest depth. It is then compressed at an 80-beat-per-minute rate. In Thumper^TM^, one connecting arm attached to the backboard directly compresses the heart on the sternum in the form of a piston motion, with oxygen as the driving force [8]. Compression is performed to the depth of 5-6 cm and the rate of 100 per minute. LUCAS^TM^ utilizes two connecting arms linked to the backboard and a suction cup to press in a piston motion, and the cup can induce active decompression [9]. When the overall chest depth is less than 18.5 cm, chest compressions are conducted at a depth of 5 cm or less.

The mechanical CPR with AutoPulse^TM^ has been known to have advantages in terms of improving the outcomes of cardiac arrest patients, compared to manual CPR. First, the AutoPulse^TM^ increases the diastolic blood pressure of cardiac arrest patients during CC compared to manual CPR, since it can continuously perform CC via band compression [16]. Nevertheless, there was insufficient evidence that this physiologic effect leads to better survival than manual CPR [16]. Second, the AutoPulse^TM^ has an additional advantage in the pre-hospital transport of cardiac arrest patients, since CC can be maintained at an appropriate depth and rate without interruption in an ambulance [17,18]. Despite the advantages of the AutoPulse^TM^, there are few reports that the AutoPulse^TM^ is superior to manual CPR [19]. In large population studies in Europe and the United States, the survival of patients who received the AutoPulse^TM^ was similar to that of patients who received manual CPR [20,21]. Another previous study additionally reported a 11.7% rate of serious organ injury occurring in mechanical CPR with AutoPulse^TM^ (manual CPR: 6.3%) [4]; organ injury included pneumothorax, liver rupture and emphysema, and fractures of multiple ribs and the sternum were accompanied in 45.6% of patients (manual CPR: 41.3%) [4]. A recent meta-analysis also reported that manual CPR has a lower risk of pneumothorax and hematoma than mechanical CPR with the AutoPulse^TM^ [10]. Our study also suggests that the in-hospital use of the AutoPulse^TM^ for mechanical CPR could significantly lower survival compared to manual CPR.

Another factor of AutoPulse^TM^ to consider is compression rate. CPR guidelines recommend a depth of compression of 100–120 per minute for high-quality CPR [4]. The compression rate of AutoPulse^TM^ is only 80 beats per minute, which is insufficient for high-quality CPR. Even when compared to other machines (Thumper and LUCAS are 100 ± 6 per minute and 102 ± 2 per minute, respectively), it is set low. This should be considered a variable that can influence the outcome as compared to manual CPR.

During mechanical CPR with Thumper^TM^, the high energy compressed by air is converted into energy that the piston can use to compress the chest, leading to an increase in cardiac output [22]. However, the high piston energy and one connecting arm of Thumper^TM^ may also cause serious organ damage and induce a change in the CC point or non-vertical CC. Lin et al. showed no significant difference in early survival for OHCA patients performing mechanical CPR with Thumper^TM^ versus manual CPR in the hospital [13].

The LUCAS^TM^ device can perform high-quality CPR by maintaining a consistent compression point using two connecting arms and by inducing active decompression via the suction cup. Previous studies have reported that LUCAS^TM^ can maintain a higher cardiac output, higher carotid blood flow, and higher cardiac perfusion pressure than manual CPR [23,24]. In a large population randomized trial of OHCA patients, such as the LINC and PARAMEDIC study, mechanical CPR with LUCAS^TM^ demonstrated the same sustained ROSC and survival rate, compared to manual pre-hospital CPR by paramedics [25,26]. In a systematic review and meta-analysis comparing LUCAS^TM^ and manual CPR, LUCAS^TM^ produced the same ROSC and survival as manual CPR [8,21,27]. Another advantage of LUCAS^TM^ is its capability of continuous CC during transport for emergency procedures, such as PCI [17,28]. In addition, mechanical CPR with LUCAS^TM^ can significantly reduce the interruption time during CC, compared to manual CPR [29]. Nonetheless, some autopsy studies have shown that LUCAS^TM^ can cause microfractures of the sternum or multiple ribs, which were not identified by post-mortem computed tomography [30]. In a multi-center study comparing manual CPR with mechanical CPR using LUCAS^TM^, the LUCAS^TM^ group showed a similar frequency of sternal fracture (LUCAS^TM^: 58.3% vs. manual CPR: 54.2%, *p* = 0.555) and a higher frequency of rib fractures than that of manual CPR (LUCAS^TM^: 78.8% vs. manual CPR: 64.6%, *p* = 0.021) [31,32].

This study has several limitations. First, this study included a wide representation of the population of South Korea, but these results may be different in studies of another race or country. Second, regardless of our efforts to adjust for confounding factors using PSM and multivariate analysis, the data should be carefully interpretated due to the selection bias inherent with the nature of an observational study. Third, we only analyzed short-term survival provided by the OHCAS database, since this database did not provide long-term survival metrics; therefore, the effect of mechanical CPR may differ for long-term survival. Fourth, the sample size of the mechanical CPR group was not as large as that of the manual CPR group; even though PSM was applied to resolve the imbalanced sample sizes between groups, the effect of mechanical CPR should be reassessed in future large population studies.

## 5. Conclusions

The investigated mechanical CPR devices mostly led to equal survival as that of manual CPR among OHCA patients; however, the in-hospital use of the AutoPulse^TM^ for mechanical CPR may significantly lower survival compared to manual CPR.

## Figures and Tables

**Table 1:** Comparison of the compression mechanism in three different mechanical CPR devices.

Classification		AutoPulse^TM^	Thumper^TM^	LUCAS^TM^
Specification of device	Backboard	Yes	Yes	Yes
	Connecting arm	2	1	2
	Compression band	Yes	No	No
	Compression tool	Band type	Pad type	Suction cup type
	Movement of the device during CPR	Possible	Possible	Possible
	Driving force	Battery	Oxygen or air	Battery
CC parameters	CC depth	20% of Chest AP diameter	5–6 cm	4–5 cm
	CC rate	80 ± 5/min	100 ± 6/min	102 ± 2/min
	Duty cycle	50 ± 5%	50/50 cycle	50 ± 5%
	CC location	Unknown	The lower half of the sternum	The lower half of the sternum
	Active decompression	Impossible	Impossible	Possible

Acronyms: CPR, cardiopulmonary resuscitation; CC, chest compression; and AP, anteroposterior.

**Table 2:** Comparison of the compression mechanism in three different mechanical CPR devices after propensity score matching.

Devices	AutoPulse^TM^			Thumper^TM^			LUCAS^TM^		
	Manual CPR ** N = 671	Mechanical CPR ** N = 671	*p*-Value *	Manual CPR ** N = 305	Mechanical CPR ** N = 305	*p*-Value *	Manual CPR ** N = 149	Mechanical CPR ** N = 149	*p*-Value *
Age, years	69 (57–78)	69 (57–78)	0.951	69 (56–78)	71 (56–78)	0.790	68 (56–79)	70 (56–78)	0.705
Male	436 (65.0%)	445 (66.3%)	0.646	185 (60.7%)	205 (67.2%)	0.109	96 (64.4%)	101 (67.8%)	0.624
Witnessed	380 (56.6%)	422 (62.9%)	0.022	189 (62.0%)	184 (60.3%)	0.740	94 (63.1%)	95 (63.8%)	1.000
Place			0.888			0.048			1.000
Non-public	545 (81.2%)	548 (81.7%)		258 (84.6%)	238 (78.0%)		116 (77.9%)	115 (77.2%)	
Public	126 (18.8%)	123 (18.3%)		47 (15.4%)	67 (22.0%)		33 (22.1%)	34 (22.8%)	
Bystander CPR	176 (26.2%)	191 (28.5%)	0.391	69 (22.6%)	80 (26.2%)	0.346	53 (35.6%)	53 (35.6%)	
Arrest cause			0.923			1.000			0.193
Cardiac	612 (91.2%)	614 (91.5%)		280 (91.8%)	281 (92.1%)		134 (89.9%)	141 (94.6%)	
Non-cardiac	59 (8.8%)	57 (8.5%)		25 (8.2%)	24 (7.9%)		15 (10.1%)	8 (5.4%)	
Arrest rhythm			1.000			0.506			0.561
Non-shockable	568 (84.6%)	568 (84.6%)		260 (85.2%)	253 (83.0%)		117 (78.5%)	122 (81.9%)	
Shockable	103 (15.4%)	103 (15.4%)		45 (14.8%)	52 (17.0%)		32 (21.5%)	27 (18.1%)	
PCI	10 (1.5%)	10 (1.5%)	1.000	8 (2.6%)	6 (2.0%)	0.787	12 (8.1%)	8 (5.4%)	0.487
TTM	22 (3.3%)	47 (7.0%)	0.003	9 (3.0%)	9 (3.0%)	1.000	14 (9.4%)	14 (9.4%)	1.000
Pacemaker	3 (0.4%)	0 (0.0%)	0.249	4 (1.3%)	3 (1.0%)	1.000	4 (2.7%)	1 (0.7%)	0.371
ECMO	7 (1.0%)	19 (2.8%)	0.029	6 (2.0%)	4 (1.3%)	0.750	11 (7.4%)	11 (7.4%)	1.000
Sustained ROSC	240 (35.8%)	203 (30.3%)	0.037	110 (36.1%)	62 (20.3%)	<0.001	69 (46.3%)	41 (27.5%)	0.001
Survival to discharge	42 (6.3%)	33 (4.9%)	0.342	14 (4.6%)	10 (3.3%)	0.532	8 (5.4%)	4 (2.7%)	0.377

Categorical and continuous variables are represented by a number (%) and median (interquartile range), respectively. Acronyms: CPR, cardiopulmonary resuscitation; PCI, percutaneous coronary intervention; TTM, target temperature management; ECMO, extracorporeal cardiopulmonary support; and ROSC, return of spontaneous circulation. * Calculated by Mann–Whitney test for continuous variables, and Chi-squared or Fisher’s exact test for categorical variables. ** Performed by univariate analysis after 1:1 propensity score matching.

**Table 3:** Matched multivariate analysis for three types of mechanical CPR devices with respect to sustained ROSC.

AutoPulse^TM^			Thumper^TM^			LUCAS^TM^		
Factors	aOR (95% CI) *	*p*-Value	Factors	aOR (95% CI) *	*p*-Value	Factors	aOR (95% CI) *	*p*-Value
Witnessed	2.05 (1.60–2.62)	<0.001	Witnessed	2.38 (1.57–3.60)	<0.001	Male	0.60 (0.36–1.02)	0.060
Arrest cause (cardiac origin)	0.38 (0.26–0.57)	<0.001	Arrest cause (cardiac origin)	0.29 (0.15–0.54)	<0.001	Witnessed	2.05 (1.19–3.55)	0.010
Mechanical CPR devices	0.74 (0.58–0.93)	0.011	Arrest rhythm (shockable)	1.48 (0.91–2.41)	0.115	Arrest cause (cardiac origin)	0.14 (0.05–0.41)	<0.001
			Mechanical CPR devices	0.43 (0.30–0.63)	<0.001	Mechanical CPR devices	0.45 (0.27–0.75)	0.002

Acronyms: aOR, adjusted odds ratio; CI, confidence interval; CPR, cardiopulmonary resuscitation; and ROSC, return of spontaneous circulation. * Calculated by multivariate logistic regression (stepwise backward elimination).

**Table 4:** Matched multivariate analysis for three types of mechanical CPR device with respect to patient survival at discharge.

AutoPulse^TM^			Thumper^TM^			LUCAS^TM^		
Factor	aOR (95% CI) *	*p*-Value	Factor	aOR (95% CI) *	*p*-Value	Factor	aOR (95% CI) *	*p*-Value
Witnessed	3.89 (1.95–7.73)	<0.001	Witnessed	3.53 (0.90–13.92)	0.071	Age, year	0.96 (0.92–1.00)	0.033
Arrest cause (cardiac origin)	0.50 (0.23–1.09)	0.081	Arrest rhythm (shockable)	2.92 (1.09–7.79)	0.032	Arrest cause (cardiac)	5379780.42 (0.00–)	0.990
Arrest rhythm (shockable)	2.00 (1.11–3.61)	0.021	PCI	4.24 (0.87–20.59)	0.072	TTM	6.51 (1.65–25.74)	0.007
PCI	24.60 (6.26–96.76)	<0.001	TTM	6.33 (1.75–22.98)	0.005	Pacemaker	14.20 (1.48–136.61)	0.021
TTM	15.98 (8.41–30.37)	<0.001	Pacemaker	15.95 (2.33–109.00)	0.004			
Mechanical CPR devices	0.57 (0.33–0.96)	0.035						
ECMO	0.03 (0.00–0.34)	0.004						

Acronyms: aOR, adjusted odds ratio; CI, confidence interval; CPR, cardiopulmonary resuscitation; ECMO, extracorporeal cardiopulmonary oxygenation; PCI, percutaneous coronary intervention; and TTM, target temperature management. * Calculated by multivariate logistic regression (stepwise backward elimination).

## Data Availability

The datasets generated during the current study are available from the corresponding author on reasonable request.

## Supplementary Materials

The following supporting information can be downloaded at: https://www.mdpi.com/article/10.3390/medicina58030353/s1. Table S1: Unmatched univariate analysis for three types of mechanical CPR devices.
