# Supplementary material for: Prognostic Impact of In-Hospital Use of Mechanical Cardiopulmonary Resuscitation Devices Compared with Manual Cardiopulmonary Resuscitation: A Nationwide Population-Based Observational Study in South Korea
Source: Medicina (Kaunas). 2022 Feb 27;58(3):353. doi: 10.3390/medicina58030353 (PMC8954998; doi:10.3390/medicina58030353)
Supplement: Supplementary file 1 [file medicina-58-00353-s001.zip › medicina-1603659-supplementary.pdf]

Supplementary Materials

Table S1. Unmatched univariate analysis for three types of mechanical CPR devices.

Table S1. Unmatched univariate analysis for three types of mechanical CPR devices.

Devices	AutoPulse™			Thumper™			LUCAS™		
	**Manual CPR N = 19,045**	**Mechanical CPR N = 671**	***p*-value***	**Manual CPR N = 19,045**	**Mechanical CPR N = 305**	***p*-value***	**Manual CPR N = 19,045**	**Mechanical CPR N = 149**	***p*-value***
Age, years	67.0 (55.0-77.0)	69.0 (57.0–78.0)	0.016	67.0 (55.0-77.0)	71.0 (56.0-78.0)	0.099	67.0 (55.0-77.0)	70.0 (56.0-78.0)	0.249
Male	12,911 (67.8%)	445 (66.3%)	0.447	12,911 (67.8%)	205 (67.2%)	0.878	12,911 (67.8%)	101 (67.8%)	1.000
Witnessed	11,725 (61.6%)	422 (62.9%)	0.513	11,725 (61.6%)	184 (60.3%)	0.703	11,725 (61.6%)	95 (63.8%)	0.643
Place			0.349			0.403			0.427
Non-public	15,260 (80.1%)	548 (81.7%)		15,260 (80.1%)	238 (78.0%)		15,260 (80.1%)	115 (77.2%)	
Public	3,785 (19.9%)	123 (18.3%)		3,785 (19.9%)	67 (22.0%)		3,785 (19.9%)	34 (22.8%)	
Bystander CPR	4,814 (25.3%)	191 (28.5%)	0.069	4,814 (25.3%)	80 (26.2%)	0.754	4,814 (25.3%)	53 (35.6%)	**0.005**
Arrest cause			0.577			0.483			0.141
Cardiac	17,292 (90.8%)	614 (91.5%)		17,292 (90.8%)	281 (92.1%)		17,292 (90.8%)	141 (94.6%)	
Non-cardiac	1,753 (9.2%)	57 (8.5%)		1,753 (9.2%)	24 (7.9%)		1,753 (9.2%)	8 (5.4%)	
Arrest rhythm			0.010			0.330			0.763
Non-shockable	15,343 (80.6%)	568 (84.6%)		15,343 (80.6%)	253 (83.0%)		15,343 (80.6%)	122 (81.9%)	
Shockable	3,702 (19.4%)	103 (15.4%)		3,702 (19.4%)	52 (17.0%)		3,702 (19.4%)	27 (18.1%)	
PCI	650 (3.4%)	10 (1.5%)	0.009	650 (3.4%)	6 (2.0%)	0.221	650 (3.4%)	8 (5.4%)	0.280
TTM	1,173 (6.2%)	47 (7.0%)	0.417	1,173 (6.2%)	9 (3.0%)	**0.028**	1,173 (6.2%)	14 (9.4%)	0.143
Pacemaker	187 (1.0%)	0 (0.0%)	0.017	187 (1.0%)	3 (1.0%)	1.000	187 (1.0%)	1 (0.7%)	1.000
ECMO	389 (2.0%)	19 (2.8%)	0.203	389 (2.0%)	4 (1.3%)	0.488	389 (2.0%)	11 (7.4%)	**< 0.001**
Sustained ROSC	7,606 (39.9%)	203 (30.3%)	<0.001	7,606 (39.9%)	62 (20.3%)	**< 0.001**	7,606 (39.9%)	41 (27.5%)	**0.003**
Survival to discharge	1,376 (7.2%)	33 (4.9%)	0.028	1,376 (7.2%)	10 (3.3%)	**0.011**	1,376 (7.2%)	4 (2.7%)	**0.048**

Categorical and continuous variables are represented by a number (%) and median (interquartile range), respectively. Acronyms: CPR, cardiopulmonary resuscitation; PCI, percutaneous coronary intervention; TTM, target temperature management; ECMO, extracorporeal cardiopulmonary support; and ROSC, return of spontaneous circulation. *Calculated by Mann–Whitney test for continuous variables, and Chi-squared or Fisher's exact test for categorical variables.
